# The Breast Tumor Microenvironment: A Key Player in Metastatic Spread

**DOI:** 10.3390/cancers13194798

**Published:** 2021-09-25

**Authors:** Lucas E. L. Terceiro, Chidalu A. Edechi, Nnamdi M. Ikeogu, Barbara E. Nickel, Sabine Hombach-Klonisch, Tanveer Sharif, Etienne Leygue, Yvonne Myal

**Affiliations:** 1 Department of Pathology, Max Rady College of Medicine, University of Manitoba, Winnipeg, MB R3E 3P5, Canada; evangel4@myumanitoba.ca (L.E.L.T.); edechia@myumanitoba.ca (C.A.E.); tanveer.sharif@umanitoba.ca (T.S.); 2 Department of Immunology, Max Rady College of Medicine, University of Manitoba, Winnipeg, MB R3E 0T5, Canada; ikeogun@myumanitoba.ca; 3 Institute of Cardiovascular Sciences, St. Boniface Hospital Albrechtsen Research Centre, Winnipeg, MB R2H 2A6, Canada; BNickel@sbrc.ca; 4 Department of Human Anatomy and Cell Sciences, Max Rady College of Medicine, University of Manitoba, Winnipeg, MB R3E 0J9, Canada; sabine.hombach@umanitoba.ca; 5 Department of Biochemistry and Medical Genetics, Max Rady College of Medicine, University of Manitoba, Winnipeg, MB R3E 0T5, Canada; etienne.leygue@umanitoba.ca; 6 Senior Scientist, CancerCare Manitoba Research Institute, Winnipeg, MB R3E 0V9, Canada; 7 Department of Physiology and Pathophysiology, Max Rady College of Medicine, University of Manitoba, Winnipeg, MB R3E 0J9, Canada

**Keywords:** breast cancer, tumor microenvironment, metastasis, exosomes, circulating tumor cells, prolactin inducible protein

## Abstract

**Simple Summary:**

The spread of breast cancer to distant parts of the body (metastasis) is the major cause of death in breast cancer patients. Research has shown that apart from the breast cancer cells themselves, other cells and molecules in the vicinity (the tumor microenvironment) also greatly contribute to the ability of breast cancer to metastasize. In this review article, we discuss recent advances in research about how breast cancer cells interact with other cells and molecules around them. We also highlight some new technologies to further unravel the nature of this interaction and how this could be beneficial in developing more effective treatments for advanced breast cancer patients.

**Abstract:**

The tumor microenvironment plays a pivotal role in the tumorigenesis, progression, and metastatic spread of many cancers including breast. There is now increasing evidence to support the observations that a bidirectional interplay between breast cancer cells and stromal cells exists within the tumor and the tumor microenvironment both at the primary tumor site and at the metastatic site. This interaction occurs through direct cell to cell contact, or by the release of autocrine or paracrine factors which can activate pro-tumor signaling pathways and modulate tumor behavior. In this review, we will highlight recent advances in our current knowledge about the multiple interactions between breast cancer cells and neighboring cells (fibroblasts, endothelial cells, adipocytes, innate and adaptive immune cells) in the tumor microenvironment that coordinate to regulate metastasis. We also highlight the role of exosomes and circulating tumor cells in facilitating breast cancer metastasis. We discuss some key markers associated with stromal cells in the breast tumor environment and their potential to predict patient survival and guide treatment. Finally, we will provide some brief perspectives on how current technologies may lead to the development of more effective therapies for the clinical management of breast cancer patients.

## 1. Introduction

### 1.1. Breast Cancer: Current Challenges

Breast cancer (BC) affects more than 2.1 million women worldwide each year and accounts for approximately 15% of all cancer-related deaths [1]. It is estimated that 6 to 10% of women diagnosed with BC have stage 4 metastatic BC, and ~85% of them will not survive [2]. Despite significant advances in the treatment of BC, metastatic relapse remains a major challenge for patients, clinicians and breast cancer researchers. These challenges have now been further exacerbated by the coronavirus (COVID-19) pandemic. Consultations and treatment of BC patients are delayed [3], providing an ideal situation for tumor progression and metastasis, thereby negatively impacting BC patients’ overall survival. Thus, an in-depth understanding of the factors that drive BC metastasis is critical in developing novel therapeutic strategies against the disease.

### 1.2. Breast Cancer Subtypes

Breast cancer is a very heterogeneous disease. As such, pathologists have defined many subtypes based on histological features. The majority of tumors belong to the invasive ductal carcinoma histological subtype (approximately 70 to 80% of newly detected tumors). The remainder are known as special types, such as lobular carcinomas (the most common special type representing 5% to 15% of newly detected cases), mucinous carcinomas (2%) and tubular carcinomas (2%) [4].

Specific markers have also been used to subclassify breast cancers. The markers most frequently used in the clinic are the receptors for estrogen and progesterone (ER, PR) as well as the human epidermal growth factor receptor 2 (HER2) (also known as ErbB2 and Neu). The expression of these markers has prognostic value. For example, ER+/PR+ tumors have a better prognosis than ER-/PR- tumors, and HER2+ tumors are more aggressive than HER2− tumors. Tumors that do not express ER, PR or HER2 are known as triple-negative breast cancer (TNBC).

Global gene expression profiling has further led to the identification of additional molecular subtypes; luminal A, luminal B, basal-like, normal-like and more recently, claudin-low tumors [5,6,7,8]. Of course, there is overlap between classification systems. For example, ER+ are mostly luminal A, or luminal B (these typically show lower PR expression and are more proliferative than luminal A tumors). Additionally, many TNBC are basal-like. A distinct classification system has been developed based on integrating genomic level changes together with gene expression signatures. This approach has led to the development of 10 integrative clusters [9,10].

Yet another tier of classification focused on defining four distinct molecular TNBC subtypes (BL1, BL2, M and LAR) has been described [11,12]. TNBC are aggressive and hard to treat BC, often displaying chemoresistance and increased distant recurrence with more frequent relapses and a higher incidence of metastasis to the brain [12]. As a consequence, these patients have a worse prognosis and higher recurrence rate when compared to other subtypes [12].

Importantly, the route of metastatic dissemination is very much affected by histological and molecular subtypes [13]. Thus, the identification and characterization of more clinically distinct molecular subtypes of BC will assist not only in more accurately assessing the probability of BC metastasis, but also in predicting possible metastatic sites as well [14,15].

### 1.3. The Tumor Microenvironment

The course of BC progression is not only determined by the specific subtype of BC or genomic events within tumor cells, but also by the composition of the tumor microenvironment (TME) [16]. Tumors are composed of tumor parenchyma, stromal cells and inflammatory mediators [17,18]. The stromal composition of the primary tumor is considered to be one of the most important factors dictating breast cancer progression. The complex and multilayered crosstalk between BC cells and stromal cells within the microenvironment, such as tissue-resident and peripherally recruited immune cells, fibroblasts, endothelial cells, among others, greatly influence the progression of BC [19]. As a direct result of such complexity and to further optimize therapeutic strategies for BC, it is crucial to consider not only the TME, but also cell-intrinsic and -extrinsic mediators, both in the primary tumor and metastatic sites.

## 2. Breast Cancer Cell-Stromal Interactions

### 2.1. Primary Site

In the primary breast TME, stromal cells are in constant contact with both BC cells and their secreted factors [16]. The effects of these interactions include changes in gene expression (such as metabolic reprogramming and epithelial-mesenchymal transition, EMT) not only in the BC cells, but also in all neighboring stromal cells. Changes occurring within the primary breast TME collectively favor both BC cell survival and metastasis [20]. Even though it is beyond the scope of this review to highlight the progress made in our understanding of the ways used by cancer cells and surrounding “normal” cells to communicate, a special mention should be made here about tunneling nanotubes.

#### 2.1.1. Tunneling Nanotubes

Tunneling nanotubes are cytoplasmic extensions that can reach several hundred micrometers and connect animal cells to one another [21,22]. These structures, observed in multiple different cell systems both in vivo and in vitro, allow the “horizontal transportation” of cellular content, including miRNAs, proteins, vesicles, autophagosomes and even mitochondria [22,23,24]. Interestingly, tunneling nanotubes were also found in vivo to provide the framework of a resistant network of brain tumor cells [25], emphasizing the ability of the structures to transport molecules critical for cell survival under therapy and their direct contributions to the cell-cell communication between cancer cells and tumor microenvironment (for recent review [26]). One could further extrapolate that these open connections between cancer cells and surrounding “normal” cells can blur the identity of specific cells involved in the tumor network, making it difficult to generate a targeted immune response, and by the same token, increasing the capacity of tumors to resist radiation and chemotherapy.

Even though the exact mechanisms remain unknown, cancer cells were found to actively communicate with multiple cell types within the TME, including fibroblasts, endothelial cells, adipocytes and immune cells.

#### 2.1.2. Fibroblasts

Fibroblasts constitute one of the most abundant cell types in the stroma of primary breast tumors and have been shown to enhance tumor progression and metastasis by promoting cancer cell growth, pro-tumor immune responses, extracellular matrix (ECM) remodeling and angiogenesis (development of new blood vessels) [27]. Although mainly derived from resident fibroblasts [28], BC-associated fibroblasts (BCAFs) can also originate from other cell types such as mesenchymal stem cells, cancer cells, cancer stem cells, and endothelial cells through a process known as trans-differentiation [20]. The exposure of mesenchymal stem cells to the proinflammatory cytokines, tumor necrosis factor-alpha (TNF-α) and interleukin-1 beta (IL-1β) can lead to the development of the cancer-associated fibroblast (CAF) phenotype [29]. These CAFs in turn secrete inflammatory factors and chemokines which can enhance BC migration [29]. Moreover, when BC cells are in constant interaction with normal fibroblasts, these hitherto “normal fibroblasts” permanently transition to CAFs [28,30]. Other factors expressed by BC cells such as osteopontin, drive the differentiation of mesenchymal stem cells to fibroblasts in a process mediated by integrin-dependent transforming growth factor-beta 1 (TGF-β1) expression [31].

In general, CAFs secrete major cytokines including TGF-β1, C-X-C motif chemokine ligand 12 (CXCL12), platelet-derived growth factor (PDGF) and interleukin-6 (IL-6) which promote tumor growth and metastasis [20]. Interestingly, these cytokines also enhance the formation of CAFs from other cell types in the TME via trans-differentiation [20]. BCAFs secrete factors which stimulate BC cells to gain the more aggressive mesenchymal phenotype and turn on pathways that collectively enable BC cells to escape from the primary tumor site and metastasize to distant organs [30]. Studies have also implicated fibroblasts as a major contributor to senescence-associated secretory phenotype [32] in that fibroblasts release molecules that promote the establishment of BC stem cells, enhance chemoresistance and increase the metastatic potential of BC cells [20,32].

#### 2.1.3. Endothelial Cells

Within the primary tumor, endothelial cells (ECs) are morphologically and functionally heterogeneous [33]. A subset of ECs, tumor endothelial cells (TEC), play a central role in breast cancer development and progression [33]. The interaction between BC cells and TECs can trigger angiogenesis, as well as modulate immune responses in the primary breast TME [34,35]. The tumor vasculature supplies nutrients and oxygen required for tumor growth and acts as entry points for BC cells into the systemic circulation. Therefore, through angiogenesis, cancer cells can survive the harsh tumor environment and increase their chances of spreading to distant sites [33]. The hypoxic tumor environment and factors secreted by tumor cells have been reported to stimulate pro-tumorigenic features in endothelial cells in the TME [35]. In addition to angiogenesis, recent studies have demonstrated that endothelial cells also provide signals that direct the behavior of neighboring cancer cells in the TME [36]. For instance, using in vivo and in vitro experiments, it was demonstrated that the contact between BC cells and endothelial cells enhanced mesenchymal characteristics in the endothelial cells. Resultantly, these endothelial cells with mesenchymal characteristics promoted a more aggressive breast tumor phenotype with increased proliferation, invasion and stem cell-like properties [37]. A growing body of evidence also suggests that TECs exhibit particular phenotypic and functional features compared to their normal counterparts [33]. It has been demonstrated that TECs have elevated levels of the stem cell marker, aldehyde dehydrogenase (ALDH), increasing both proangiogenic features and drug resistance [38]. TECs isolated from metastatic tumors also show up-regulation of several angiogenesis-related genes (VEGFR-1, VEGFR-2, and VEGF), as well as up-regulation of gelatinases/collagenases IV MMPs (MMP-2 and MMP-9) [39,40]. These up-regulated genes can promote breast cancer progression and metastasis.

#### 2.1.4. Adipocytes

In the TME, normal adipocytes are differentiated into cancer-associated adipocytes (CAAs) by BC cells. These CAAs then secrete chemokine C-C motif ligand 2 (CCL2), chemokine C-C motif ligand 5 (CCL5), IL-1β, IL-6, TNF-α, vascular endothelial growth factor (VEGF) and leptin, to promote the invasion and metastasis of BC cells [41,42,43,44]. The release of CCL2 by CAAs has been shown to increase adipocyte and macrophage recruitment to the TME [45]. As well, BC cells can upregulate IL-6 expression in adipocytes, which in turn promotes angiogenesis, tumor cell proliferation and survival via the Janus kinase/signal transducer and activator of transcription 3 (JAK/STAT3) signaling pathway [46]. Additionally, IL-6 induces the production and maintenance of BC stem cells through the activation of nuclear factor kappa-light-chain-enhancer of activated B cells (NF-kB) and STAT3, thereby promoting tumor progression [47]. Studies have also shown that leptin produced by CAAs can enhance BC growth by activating the JAK/STAT3 and phosphatidylinositol 3-kinase- protein kinase B (PI3K-AKT) signaling pathways in BC cells [48]. A recent study demonstrated that adipocytes can also increase proliferation and BC stem cell properties through the release of adipsin, an adipokine secreted by adipose tissue in the breast [49].

Interestingly, CAAs have been shown to provide high-energy metabolites, such as pyruvate, lactate, ketone bodies, and fatty acids, to BC cells [50,51]. To meet the extreme energy demands of dividing cells, BC cells also develop a dynamic interaction with CAAs that reprogram the metabolic process to support BC proliferation through the release of monocarboxylates [45]. In addition, adipocytes have been shown to promote mitochondrial metabolism in BC cells by downregulating caveolin 1, which increases the secretion of pyruvate and lactate by adipocytes that are taken up by BC cells and CAFs to use as an energy source [52,53].

#### 2.1.5. Immune Cells

Another major component of the primary breast TME are immune cells which can elicit both pro- and anti-tumor activity [54]. Immune cells present in the breast TME are diverse with the most common being macrophages, an innate immune cells [20]. Macrophages play dual roles in the breast tumor environment depending on whether the macrophage is classically (M1) or alternatively (M2) activated [55]. M1 macrophages produce proinflammatory cytokines with antitumor activity which contribute to BC clearance [56], whereas M2 macrophages produce anti-inflammatory cytokines supporting BC progression [55,57]. Tumor-associated macrophages (TAMs) which are often M2 macrophages, express distinctive cell surface markers (CD163, Fc fragments of IgG, C-type lectin domains, heat shock proteins). TAMs have been reported to promote the escape of primary tumor cells into the circulation by secreting epidermal growth factor-1 (EGF-1), TGF-β, IL-6, IL-10, and TNF-α, which promotes EMT, and enhances the stemness of cancer cells, ultimately increasing invasiveness and migration into surrounding vasculature (for review, [58,59]). As well, breast tumors can also avoid immune-mediated elimination, by stimulating M2 macrophage formation or upregulating programmed cell death-1 ligand (PD-L1) expression [60]. In the latter scenario, the binding of PD-L1 secreted by tumor cells to the PD-1 receptors of activated lymphocytes T leads to inactivation of cytotoxic T cells. Similar to macrophages, neutrophils have been reported to inhibit and promote metastasis depending on whether the neutrophil has the anti-tumor (N1) or pro-tumor (N2) phenotype [61,62]. Neutrophils have also been shown to promote EMT and drug resistance in cancer cells, thereby promoting metastasis [63,64]. A subset of innate immune cells, referred to as myeloid-derived suppressor cells (MDSCs) also contribute to metastasis in BC by secreting factors such as interleukin-10 (IL-10), TGF-beta and VEGF which suppress immune responses, increase EMT and stimulate angiogenesis respectively [65]. It has been shown that chemokines (such as chemokine C-C motif ligand 3, CCL3), produced by BC cells attract MDSCs to the primary tumor site, which in turn activate EMT in BC cells enhancing breast tumor invasion [66].

Regulatory T and B lymphocytes are the major adaptive immune cell subsets which dampen the anti-tumor immune response in the TME, thereby promoting tumor progression [31]. Regulatory T cells favor BC progression by sustaining pro-tumor (M2) macrophage survival [67]. In a model of metastatic BC, extensive lung metastasis was associated with increased numbers of regulatory T cells in the primary tumor, suggesting their role in enhancing BC progression [68]. Furthermore, in a mouse model, the presence of regulatory T lymphocytes in the primary tumor was associated with increased apoptosis of anti-tumor cytotoxic T lymphocytes [69]. Similar to their T cell counterparts, regulatory B cells have been shown to further increase breast tumor metastasis [70]. Another subset of T lymphocytes, the type 2 helper CD4^+^ T lymphocytes, have also been reported to promote BC progression and metastasis by stimulating epidermal growth factor signaling in BC cells and switching the activation status of tumor-associated macrophages from M1 to M2 [71].

Recently, immunotherapy approaches have been developed to treat BC patients. These therapies utilized antigen-specific monoclonal antibodies, checkpoint inhibitors and adoptive transfer of autologous lymphocytes [72,73]. In a recent phase 3 trial study, the utilization of anti-PD-L1 antibody atezolizumab, in combination with nab-paclitaxel to treat patients with advanced TNBC, resulted in prolonged progression-free survival and overall patient survival [74]. In addition, an important study demonstrated for the first time the complete durable regression of metastatic BC using adoptive transfer of autologous lymphocytes in conjunction with IL-2 and checkpoint blockade [75]. Such studies as these demonstrate that the development of BC immunotherapies can provide a broader range of effective therapies for BC patients.

### 2.2. The Metastatic Process and Preferred BC Metastatic Sites

#### 2.2.1. Metastatic Process

The metastatic process is a complex cascade of events facilitating the spread of cancer cells from the primary tumor site to distal organs. Proposed as the “seed and soil” hypothesis of cancer metastasis by Paget [76], cancer cells (seed) are thought to thrive at distant sites that provide favorable conditions (soil) where they prepare and alter the metastatic environment to ensure their survival. The metastatic process is believed to occur in 3 phases: invasion, intravasation and extravasation. During the invasion phase, the BC cells at the primary tumor site acquire aggressive features [63], which enables them to invade the basement membrane and escape the primary site.

*Epithelial-to-Mesenchymal transition (EMT):* One means by which BC cells acquire aggressive features is through the activation of EMT. EMT is a process by which epithelial BC cells acquire mesenchymal characteristics which confer a migratory phenotype and stem cell-like properties [77]. However, although previously thought to be a binary event where cancer cells acquire either epithelial or mesenchymal phenotypes, recent studies demonstrate that EMT is more complex. Some cancer cells have now been shown to possess a hybrid of epithelial and mesenchymal phenotypes which exhibit higher metastatic potential and chemoresistance compared to either “fully” epithelial or mesenchymal cancer cells (for review [78,79]). TGF-β, released by TAMs and CAFs in the primary TME has been shown to play a key role in inducing EMT. Once released, the TGF-β in turn upregulates the expression of key transcription factors, Twist, Snail and Slug, which stimulate the EMT [63]. With the newly acquired mesenchymal phenotype, the cancer cells are now able to penetrate the blood vessel (intravasation) and enter the circulation [63].

*Circulating tumor cells (CTCs):* Following intravasation, the BC cells are referred to as circulating tumor cells, CTCs. CTCs are defined as a heterogenous group of tumor cells, that are shed from the primary tumor and enter the bloodstream. However, these cells are phenotypically and genetically distinct, and generally express EMT and stem-like markers (EpCAM^+^, CK8^+^, Twist1^±^, Akt2^±^, PI3Kα^±^, CD45^-^, ALDH1^±^, CD44^high^/CD24^low^) [80,81]. CTCs can be utilized as a diagnostic marker for metastatic breast cancer. In fact, liquid biopsy with genomic and proteomic analysis has been considered a promising tool that will allow a clinical oncologist to determine the most suitable BC therapy. BC cells have been shown to migrate into the circulation either as single cells or as multicellular aggregates (referred to as collective migration) with the latter reported to possess a higher metastatic potential [82,83] than the single cells. Factors expressed by BC cells such as plakoglobin [83] and claudin 1 (for review, [84]) are reported to play key roles in facilitating their collective migration. The increased metastatic potential of multicellular aggregates is attributed to their ability to interact with stromal cells and survive the high-shear conditions in the circulation [85,86,87]. Upon arrival at the secondary site, BC cells then leave the vasculature (extravasation) and enter the distal site where they may remain as dormant cells to better adapt to the new microenvironment, or re-initiate secondary tumor growth when conditions become favorable [63].

*Mesenchymal-to-Epithelial transition (MET):* Following extravasation, BC cells may revert from a mesenchymal to epithelial phenotype by activation of MET, facilitating the invasion of secondary organs [88]. Accumulating evidence suggests that MET may play a critical role in metastatic colonization by reactivating important cell signaling pathways and enabling attachment and interaction with cells/ECM within the host tissue [89]. When BC cells arrive in secondary organs, MET is activated, and BC cells start to re-express epithelial markers, such as E-cadherin, occludin and crumbs3, and down-regulate mesenchymal transcription factors [90]. This orchestrated intracellular process provides the necessary cellular machinery required for metastatic outgrowth [91].

*Exosomes:* Interestingly, it has been suggested that exosomes, small extracellular vesicles shown to contain proteins, DNA, and RNA as well as miRNAs, directly facilitate the targeting of specific organs [92,93,94,95,96]. Seminal work performed in the laboratory of David Lyden [93] elegantly established that exosomes from cancer cells known to induce metastases in specific organs, accumulated preferentially in these same organs following injection into mouse bloodstream. These authors further demonstrated that the contents of these vesicles not only provided specific organotropism to the vesicles themselves, but also established a pre-metastatic niche able to attract tumor cells, even though these cells did not originally colonize these specific sites. The amount of specific integrins within the exosomes appeared to be responsible for an increase in S100 genes expression within target cells and ultimately in the organotropism observed. Other molecules contained in the exosome were also found to participate. For example, it was observed that exosomal miRNAs could modify the transcriptome in targets cells [97], increase trans-endothelial migration [96,98,99,100] and even promote the proliferation of osteoclasts [100]. Additionally, exosomal proteins can contribute to angiogenesis [101,102,103], disruption of the vascular barrier [104,105] and colonization of specific tissues [92,106]. As such, it appears that, through the use of exosomes, cancer cells from the primary tumor have the ability to “prepare” and “mark” the way for a successful invasion by the circulating tumor cells [107,108,109]. Interestingly, exosome content is currently being investigated as a possible new biomarker for multiple cancers [110].

#### 2.2.2. Preferred BC Metastatic Sites

##### Bone

The bone is the most common site for BC metastasis in patients [111]. Around 60 to 85% of women diagnosed with metastatic BC harbor bone metastases [112]. The blood vessels in the bone marrow are specially arranged with fenestrated endothelia, called sinusoids to allow the circulation of hematopoietic cells. As a result, the bone marrow sinusoids are more permissive to circulating tumor cells compared to other types of capillaries [113]. In addition, bone matrix cells such as osteoblasts secrete chemoattractants such as CXCL12, osteopontin, receptor activator of nuclear factor kappa-Β ligand (RANKL), and bone morphogenetic proteins (BMPs) that recruit cancer cells to the bone marrow [114,115,116]. Following their exit from the blood vessels into the bone marrow, cancer cells can then “hijack” osteoblast activity to promote osteoclastogenesis, increasing bone resorption [19]. This process results in the release of soluble factors from the bone matrix including insulin-like growth factor 1 (IGF1) and CXCL12, which stimulate activation of the PI3K-AKT pathway thereby enhancing BC cell proliferation and survival in the bone [117,118].

The release of these growth factors during osteolytic metastasis also results in a positive feedback loop between cancer cells and osteoblasts [119,120]. Pre-clinical models have shown that inhibition of TGF-β pathway reduces BC bone metastasis formation [121,122]. An additional mechanism that promotes BC bone metastasis is through the Notch ligand Jagger1 (JAG1), also modulated by TGF-β [123]. JAG1 was found to be overexpressed in TNBC and associated with metastatic relapse in the bone [123]. Recently, in vitro 3D models, which may provide new insights into the biology of BC bone metastasis, have been reported [124,125].

##### Lung

The lung is also a common metastatic site for BC [126]. The lung has a large surface area and numerous capillaries which facilitate the extravasation process and its colonization by metastatic BC cells [126]. Although the endothelial layer and basement membrane in lung capillaries are not easily permeable, BC cells can overcome this barrier by inducing vascular hyperpermeability via increased focal adhesion kinase (FAK), E-selectin and matrix metalloproteinase (MMP)-9 expression in lung endothelial cells [127,128]. As well, other factors secreted by BC cells such as cyclooxygenase 2 (COX-2), epiregulin, MMP-1 and MMP-2, contribute to increasing trans-endothelial passage to foster metastasis [129].

Once extravasation in the lung has occurred, interaction between cancer cells and lung stroma can enhance BC survival [130]. There, BC cells have been shown to secrete α6β4- and α6β1-positive exosomes which stimulate S100 calcium-binding protein A4 (S100A4) expression in the host fibroblasts to create a pre-metastatic microenvironment for tumor establishment [93]. Furthermore, BC cells secrete extracellular vesicles enriched with annexin A6 that promotes NF-kB-dependent endothelial activation, CCL2 induction, and monocyte expansion, which ultimately facilitates lung metastasis [131]. Host lung fibroblasts also build a pro-metastatic niche in the lungs by secreting TGF-β and CXCL12 which activate EMT in BC cells. Additionally, CAFs can directly induce BC cell invasion through NOTCH signaling [18]. Besides regulating BC cells, CAFs also secrete ECM proteins, such as fibronectin to recruit VEGFR1 and integrin α4β1-positive bone marrow hematopoietic cells to the lungs to provide a more permissive environment for incoming BC cells [132]. Alongside fibroblasts, BC cells can recruit circulating monocytes to the lungs through tropic CCL-2 [19]. In the lungs, monocytes are then differentiated into TAMs which support tumor extravasation by releasing VEGF and IL-1β. Collectively, these result in a systemic inflammatory cascade and neutrophil-mediated promotion of BC metastasis [56,133,134].

##### Liver

A frequent site also for BC metastasis is the liver, and if left untreated can result in mortality for patients in less than a year [135]. Liver metastasis occurs in 32% of HER2+ BC patients [112]. To facilitate colonization of the liver, BC cells can enhance their ability to attach to liver endothelium by secreting proinflammatory cytokines [136]. Chemokines and chemokine receptor molecules such as CCL2 and CXCR4, respectively have also been shown to promote BC liver metastasis by enhancing the recruitment of cancer cells and TAMs to the liver [137,138]. The resident cells in the liver can also promote the formation of a pro-metastatic niche. Studies have shown that hepatic stellate cells release growth factors and cytokines, such as hepatocyte growth factor (HGF), TGF-β and PDGF which promote ECM remodeling and angiogenesis, thereby establishing a favorable microenvironment for disseminating BC cells [139]. BC cells can also modulate hepatocytes to increase liver metastasis. Claudin 2, a tight junction protein, was found to be highly expressed by BC cells which have metastasized to the liver. Interestingly, further studies demonstrated that claudin 2 plays a role in BC liver metastasis by switching from a tight junction function to promoting the attachment of BC cells to hepatocytes [140].

##### Brain

Brain metastasis occurs in approximately 50% of patients with TNBC, and 33% or 14% for HER2+ and hormonal receptor-positive BC respectively [112]. BC brain metastasis has a particularly poor prognosis with high morbidity and mortality [141]. The central nervous system is protected by the blood-brain barrier (BBB), which distinguishes it from other organs. This BBB consists of non-fenestrated endothelium joined by tight junctions, astrocytes and pericytes [142]. An important step in BC metastasis to the brain is the extravasation of cells through the BBB. To cross the BBB and access the brain parenchyma, BC cells must employ specialized mechanisms such as the production of VEGF [143], MMPs [144] and cathepsin S [145], which loosen the endothelial tight junctions in the BBB.

The brain parenchyma is primarily composed of neurons and glial cells (astrocytes, microglia, and oligodendrocytes) [146]. Astrocytes, the most abundant cell type in the brain environment, support neurons by secreting growth factors and cytokines [76]. However, BC can also stimulate astrocytes to secrete IL-6, TGF-β and IGF-1, resulting in the activation of pathways that support tumor growth [147]. Additionally, BC cells can take up miR19a-enriched exosomes derived from astrocytes to reduce the expression of phosphatase and tensin homolog (PTEN), a major tumor suppressor. As well, loss of PTEN and tumor cell-induced CCL2 expression are associated with the recruitment of pro-metastatic myeloid cells [148,149]. Interestingly, previous studies showed that BC cells interact with astrocytes and activate the stimulator of interferon genes (STING) pathway, resulting in release of inflammatory cytokines which support breast tumor growth and chemoresistance [150,151].

The interactions of BC cells with other brain cells (neurons, oligodendrocytes, pericytes, microglia) still remain poorly understood. However, there is evidence to suggest that gamma-aminobutyric acid (GABA) secreted by neurons can generate reduced nicotinamide adenine dinucleotide (NADH) which supports the growth of metastatic BC in the brain [152]. Additionally, microglia, which are resident macrophages in the brain, have been shown to promote BC brain metastasis in a process that is modulated by the wingless-related integration site (Wnt) signalling pathway [153].

**Figure 1:**
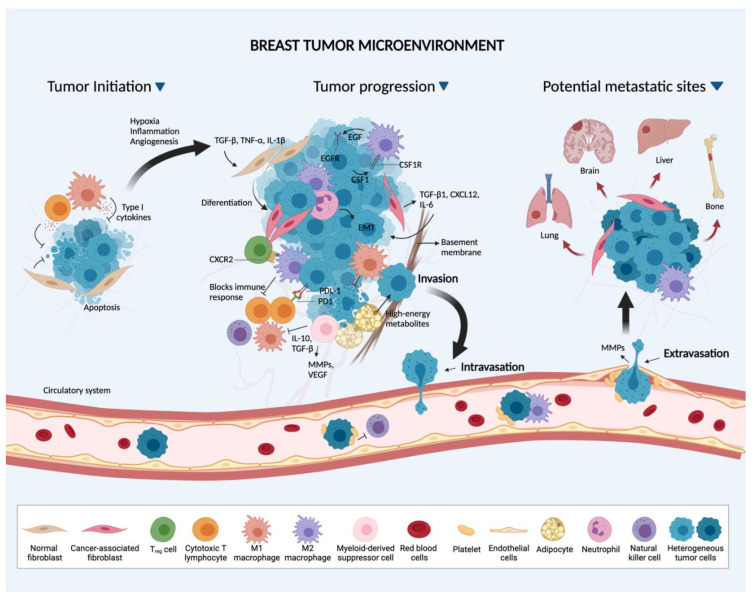
The tumor microenvironment (TME) and breast cancer progression. At the onset of tumor initiation, the developing tumor is exposed to growth-suppressive signals from the inflammatory process, which is primarily modulated by cytotoxic T-lymphocytes, M1 macrophages and fibroblasts. The BC cells overcome these mechanisms by educating host stroma cells to acquire pro-tumorigenic features. Cytokines (TGF-β, IL-1β and TNF-α) released from the inflammatory process then modulate the differentiation of normal fibroblasts to cancer-associated fibroblasts (CAFs). The latter, in turn, secrete extracellular matrix proteins and soluble factors (TGF-β, CXCL12, IL-6) that stimulate epithelial to mesenchymal transition (EMT), tumor growth and progression. Neutrophils can induce EMT and promote tumor progression through cytokines release. Adipocytes secrete high-energy metabolites to fuel tumor growth. Tumor-associated macrophages (primarily M2 macrophages) support various processes within the TME, including BC growth and invasion by secreting pro-tumorigenic cytokines and growth factors. During tumor expansion, activated cytokines in the environment (CXCL5-CXCR2, TGF-β) stimulate the recruitment of regulatory T cells (T_reg_) and myeloid-derived suppressor cells (MDSCs) which disrupt immune surveillance by inhibiting cytotoxic T lymphocytes, M1 macrophages and natural killer cells. BC cells can also escape immune surveillance by overexpressing the PD-L1 ligand. Such orchestrated events in the primary tumor allow tumor cells to acquire a mobile and invasive phenotype. Secreted factors (MMPs, VEGF) further facilitate tumor cells intravasation into the circulation. There, BC cells interact with platelets and M2 macrophages to support their survival by inhibiting immune recognition. Platelets escort tumor cells to the secondary sites, where it interacts with endothelial cells and promote extravasation. The preferred site of metastasis can be influenced by the subtype of the BC (Created with BioRender.com, accessed on 12 September 2021).

**Figure 2:**
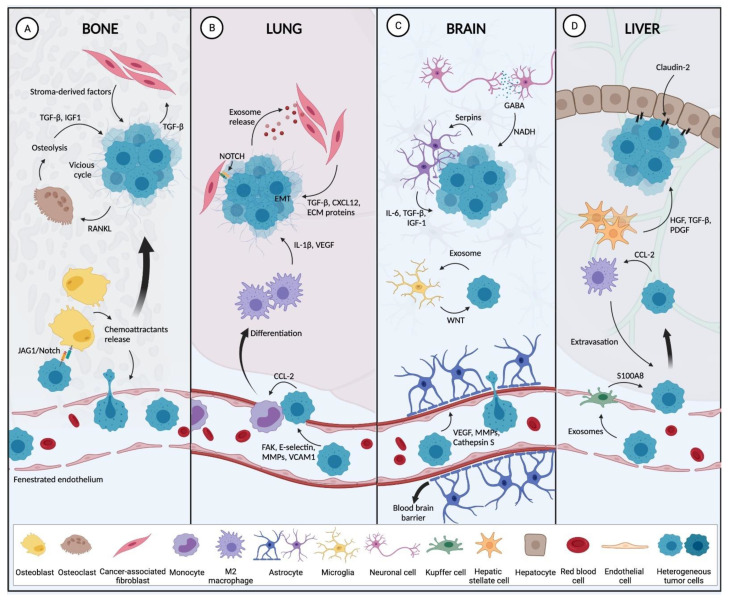
Host stromal cells at the metastatic site promote the establishment of the breast tumor. (**A**) In the bone microenvironment, chemoattractant released by osteoblasts recruit circulating tumor cells (CTCs) from the circulation to extravasate into the bone stroma. Once in the bone environment, interactions between BC cells and osteoblasts occur via JAG1/Notch and CAFs (through TGF-β), facilitating bone metastasis. There, BC cells then secrete factors to promote osteolysis, resulting in the release of factors that stimulate tumor growth, and thereby generating a vicious cycle. (**B**) In the lung capillaries, the expression of FAK, E-Selectin, VCAM1 and MMPs are involved in tumor extravasation to the lung parenchyma. Tumor cells now have the capacity to recruit monocytes from the circulation to differentiate into M2 macrophages, which then secrete pro-metastatic factors (VEGF, IL-1β). BC cells also secrete exosomes that stimulate CAF to release cytokines, growth factors and ECM components to create a pro-tumorigenic niche. (**C**) Once within the confines of the brain, tumor cells produce cathepsin S, MMPs and VEGF to overcome the blood-brain barrier in order to colonize the brain. They then stimulate astrocytes to secrete IL-6, IGF-1 and TGF-β that result in tumor expansion. Exosomes are also secreted by BC cells that stimulate microglia to support metastasis through WNT signaling. BC cells also take advantage of neurotransmitters secreted by neurons as bio-precursors to generate NADH that support tumor growth in the brain. (**D**) To promote their extravasation into the liver stroma, BC cells secrete exosomes that stimulate Kupffer cells to produce S100A8 resulting in liver-specific metastasis. BC cells also modulate M2 macrophages which also promote tumor extravasation. Hepatic stellate cells, secrete HGF, TGF-β and, PDGF to induce liver metastasis. Interaction of BC cell enriched in claudin-2 and hepatocytes also result in liver metastasis establishment (Created with BioRender.com, accessed on 12 September 2021).

Breast cancer-stromal interactions at the primary and metastatic sites are summarized in Figure 1 and Figure 2.

## 3. Breast Cancer Cell-Stromal Interactions: Implications for Prognosis

Since accumulating evidence has shown that stromal cells are key players in modulating BC cell behavior at both the primary tumor and metastatic sites, it is not surprising that these same cells greatly influence the course of BC progression. Recent studies demonstrated that the presence of specific subpopulations of CAFs in BC is strongly associated with metastatic recurrence and patient outcome [154,155]. Furthermore, microarray analysis of advanced BC patient samples revealed that CAFs exhibit metastatic site-specific protein expression profiles [156]. For example, while platelet-derived growth factor receptor A (PDGFR-α), S100A4 and podoplanin levels were elevated in bone metastasis, the levels of these proteins were lower in CAFs in metastatic liver [156].

Angiogenesis is largely mediated by endothelial cells in promoting tumor progression [34]. The pro-angiogenic factor, interleukin-3 (IL-3), released by endothelial cells was shown to promote TNBC progression when it binds to its receptor on BC cells [157]. Assessment of the number of circulating endothelial cells can also provide insight into the potential response of BC patients to therapy. For example, it has been shown that the number of circulating endothelial cells in metastatic HER2+ BC patients treated with chemotherapy in conjunction with bevacizumab (an antibody which binds VEGF) is associated with patient outcome and response to chemotherapy. However, one major limitation of such a study is that the exact time when circulating endothelial cells are measured can significantly impact the prediction [158].

The type and number of immune cells present in the breast tumor environment can also predict disease outcome or chances of survival for BC patients [159]. In particular, the number of MDSCs in breast tumors has been associated with metastasis and disease severity [160]. Accordingly, a lower frequency of MDSCs has been shown to correlate with less aggressive disease and better outcomes in patients with BC and *vice versa* [160,161]. Furthermore, BC patients who respond poorly to chemotherapy have a higher number of MDSCs compared to patients who respond better [162], while treatment with chemotherapy significantly reduced MDSC levels in BC patients [160]. As well, M2 macrophages are associated with a worse prognosis in BC patients [163].

The exact prognostic significance and clinical relevance of assessing tumor-infiltrating T and B lymphocytes in BC remain controversial, likely as a direct result of the heterogeneity of these particular subsets of immune cells [164,165]. However, some specific subsets have been shown by many studies to be associated with worse outcomes in BC patients, in that increased regulatory T cells in breast tumors were associated with shorter survival periods, poor response to therapy and worse outcomes [164,166,167].

## 4. Breast Cancer Cell-Stromal Interactions: Implications for Oncotherapy

Many approaches which target BCAFs to enhance the efficacy of BC therapies have been proposed and pursued [168]. One such example is the use of vaccines to target the fibroblast activation protein alpha (FAPα), a protein specifically expressed by BCAFs [169]. This strategy has proven to be effective in reducing BCAF numbers, inhibiting tumor growth and enhancing antitumor immune responses in mice [170,171]. Another therapeutic agent, a protein designed to target integrin αvβ3 expressed by fibroblasts, showed significant fibroblast killing activity, reduced the levels of protumor factors released by the fibroblasts and decreased tumor growth [172]. Interestingly, this therapeutic agent was also effective against endothelial cells which abundantly express integrin αvβ3 as well, and significantly prolonged survival when administered to breast tumor-bearing mice [172]. Clinical trials have shown that the antiangiogenic agent, bevacizumab (an anti-VEGF-A antibody), in combination with chemotherapy is effective against BC and promotes tumor regression and progression-free survival [173,174].

Multiple studies have also investigated the potential of targeting immune cells associated with tumor cells. Therapeutic exosomes have been used to eliminate pro-tumor M2 macrophages, leading to reduced tumor size and improved survival [175]. An artificial dual-function supramolecule (anti-SIRPα–AK750) was successfully used to inhibit CSF-1R signaling and reprogram M2 macrophages to phagocytose tumor cells [176]. Recently nanoparticles which inhibit the M1-M2 reprogramming process were shown to enhance antitumor immune response, reduce tumor growth and reduce metastasis in a mouse BC model [177]. Treatment of breast tumor-bearing mice with a fusion protein made up of diphtheria toxin and mouse IL-4 (DABIL-4) which targets cells expressing the IL-4 receptor resulted in a significant reduction in the numbers of MDSCs, TAMs and regulatory T cells. Additionally, there was notably reduced tumor growth and lung metastasis [178]

Other studies have been conducted to test potential avenues to reduce/eliminate regulatory T cell-mediated suppression of anti-tumor immune response, directly or indirectly. While some methods may directly target proteins specific to regulatory T cells (CD25; cytotoxic T-lymphocyte-associated protein 4, CTLA-4; and tumor necrosis factor receptor superfamily, member 4, TNFRSF4), it was also effective in targeting proteins and signaling molecules such as T-cell receptor (TCR) and interleukin-2 receptor (IL-2R) signaling, which are necessary for the survival and activity of regulatory T cells [179]. BC patients treated with a vaccine cocktail containing tumor-associated peptides and later treated with daclizumab (an anti-CD25 antibody) showed delayed disease progression [180]. The number of regulatory T cells in the tumor environment can also be drastically reduced by disrupting signaling pathways activated by kinases. One such example was the inactivation of p110δ (an isoform of PI3K) in regulatory T cells was shown to significantly enhance cytotoxic T lymphocyte activity and inhibit breast tumor growth [181]. Interestingly, it has also been proposed that targeting the gamma isoform of PI3K can promote antitumor immune responses [182]. As well, there are certain chemotherapeutic agents such as cyclophosphamide which preferentially eliminate regulatory T cells [183]. BC patients treated with low-dose cyclophosphamide had reduced regulatory T cells, resulting in enhanced anti-tumor immune response [184].

## 5. Conclusions and Future Perspectives

Although significant advances have been made in the past years, many facets of BC cell-stroma interactions remain unexplored. The origin and roles of the different fibroblast populations in the primary and metastatic sites currently remain poorly understood. As well, in addition to the type/nature of stromal cells and BC in the tumor environment, determining the spatial and temporal properties/dynamics of these cells in the TME is critical to better understand the metastatic process in BC. To this end, it is necessary to advance current research in the field using novel high-throughput molecular analysis and imaging techniques that provide information on the location of cells in the TME and monitor their interactions over time. Some other unique challenges about BC-stroma interactions which new techniques will help unravel include addressing why some molecules play different roles in the primary breast tumor environment versus the metastatic site. Recent studies from our laboratory have shown that the prolactin inducible protein (a breast-specific gene product), while suppressing primary tumor growth, at the same time, enhances BC metastasis in the lung [185]. A greater understanding of the mechanism of this “dual-effect” phenomenon could aid the development of therapeutic strategies which inhibit/dampen pro-tumor effects and/or amplify anti-tumor effects of certain host molecules. It is also relevant to address how the tumor environment (at the primary or metastatic site) changes during disease progression and why some BCs develop resistance to therapeutic strategies.

Single-cell RNA sequencing (scRNA-seq) is a relatively new technology which can be employed to assess changes in the TME during BC progression [186,187]. In a recent study, comparison of normal breast, precancerous breast tissue and different BC subtypes by scRNA-seq analysis showed increased presence of immune cells as normal breast tissue progressed to breast cancer [187]. As well, TNBC and HER2+ breast cancer displayed higher levels of cytotoxic T-lymphocytes compared to the luminal subtypes, indicating heterogeneity in immune response across BC subtypes [187].

Digital spatial profiling (DSP), which involves multiplex spatial profiling of proteins or RNAs from fixed patient tissue samples, is another important advancement as it can be used to assess spatial changes within the TME [188]. DSP analysis of fixed BC samples (to assess changes in the nature and location of all cells in the tumor environment before and after chemotherapy) revealed that the extent of CD45 molecule expression in the samples (especially HER2+ BC) can predict response to chemotherapy [188]. These findings underscore the importance of examining responses, pre- and post-treatment, and suggest that treatment may affect the geographical distribution of tumor cells as well as tumor cell content. Altogether, increased adoption of scRNA-seq and DSP in research and clinical practice will guide the development of more effective therapies and enable clinicians to appropriately tailor therapeutic interventions to patients based on assessments of changes to the TME and patient response to therapy.

BC cells interaction within the tumor microenvironment with either host stromal cells or with non-cellular components, such as the ECM, often result in tumor establishment and progression. Recently, new in vitro techniques have emerged to further elucidate how intercellular interactions can promote tumor progression. Three-dimensional (3D) tissue culture techniques containing various functional substrates (collagen, fibronectin, laminin, and gelatin), and multi-cellular components which recreate the tumor microenvironment more accurately have been developed [189,190]. These new in vitro techniques are valuable tools for studying tumor biology and would allow a better understanding of cellular interactions within the tumor microenvironment that could be possibly used for targeted therapy development [191,192].

In conclusion, we have summarized recent advances in our understanding of BC cell-stroma interactions at both the primary tumor and metastatic sites. We also discussed the implications of these interactions in prognosis and therapy decision-making. We then highlighted some new technologies which will not only provide more physiologically and clinically relevant insights on these interactions, but also help design new therapies and optimize patient outcomes.

## Abbreviations

ALDH1	Aldehyde Dehydrogenase 1
AKT	Protein Kinase B
BBB	Blood Brain Barrier
BC	Breast Cancer
BCAFs	Breast Cancer Associated Fibroblasts
BMPs	Bone Morphogenetic Proteins
CAA	Cancer Associated Adipocyte
CAF	Cancer Associated Fibroblast
CCL2	Chemokine C-C Motif Ligand 2
CCL3	Chemokine C-C Motif Ligand 3
CCL5	Chemokine C-C Motif Ligand 5
COX-2	Cyclooxygenase 2
CTCs	Circulating Tumor Cells
CTLA-4	Cytotoxic T-Lymphocyte-Associated Protein 4
CXCL12	C-X-C Motif Chemokine Ligand 12
DSP	Digital Spatial Profiling
EC	Endothelial Cells
ECM	Extracellular Matrix
EGF-1	Epidermal Growth Factor-1
EGFR	Epidermal Growth Factor Receptor
EMT	Epithelial-Mesenchymal Transition
ER	Estrogen Receptor
FAK	focal adhesion kinase
FAPα	Fibroblast Activation Protein Alfa
GABA	Gamma Aminobutyric Acid
HER2	Human Epidermal Growth Factor Receptor 2
HGF	Hepatocyte Growth Factor
IGF1	Insulin-like Growth Factor 1
IL-10	Interleukin-10
IL-1β	Interleukin-1 Beta
IL-3	Interleukin 3
IL-6	Interleukin-6
JAG1	Notch Ligand Jagger1
JAK/STAT3	Janus Kinase/Signal Transducer and Activator of Transcription 3
M1	Classically Activated Macrophages
M2	Alternatively activated Macrophages
MDSCs	Myeloid Derived Suppressor Cells
MMPs	Matrix Metalloproteinase
NADH	Reduced Nicotinamide Adenine Dinucleotide
NF-kB	Nuclear Factor Kappa-Light-Chain-Enhancer of Activated B Cells
PD-1	Programmed Cell Death-1
PDGF	Platelet-Derived Growth Factor
PDGFR-α	Platelet-Derived Growth Factor Receptor A
PD-L1	Programmed Cell Death-1 Ligand
PI3K	Phosphatidylinositol 3-Kinase
PR	Progesterone Receptor
PTEN	Phosphatase and Tensin Homolog
RANKL	Receptor Activator of Nuclear Factor Kappa-Β Ligand
S100A4	S100 Calcium Binding Protein A4
scRNA-seq	Single Cell RNA Sequencing
STING	Stimulator of Interferon Genes
TAM	Tumor Associated Macrophages
TEC	Tumor endothelial cells
TGF-β1	Transforming Growth Factor-Beta 1
TME	Tumor Microenvironment
TNBC	Triple-Negative Breast Cancer
TNFRSF4	Tumor Necrosis Factor Receptor Superfamily, Member 4
TNF-α	Tumor Necrosis Factor-Alpha
VEGF	Vascular Endothelial Growth Factor
Wnt	Wingless-related Integration Site
